# A Potential Prognostic Gene Signature for Predicting Survival for Glioblastoma Patients

**DOI:** 10.1155/2019/9506461

**Published:** 2019-03-26

**Authors:** Ziming Hou, Jun Yang, Hao Wang, Dongyuan Liu, Hongbing Zhang

**Affiliations:** Department of Neurosurgery, Beijing Luhe Hospital, Capital Medical University, Beijing 101149, China

## Abstract

**Objective:**

This study aimed to screen prognostic gene signature of glioblastoma (GBM) to construct prognostic model.

**Methods:**

Based on the GBM information in the Cancer Genome Atlas (TCGA, training set), prognostic genes (Set X) were screened by Cox regression. Then, the optimized prognostic gene signature (Set Y) was further screened by the Cox-Proportional Hazards (Cox-PH). Next, two prognostic models were constructed: model A was based on the Set Y; model B was based on part of the Set X. The samples were divided into low- and high-risk groups according to the median prognosis index (PI). GBM datasets in Gene Expression Ominous (GEO, GSE13041) and Chinese Glioma Genome Atlas (CGGA) were used as the testing datasets to confirm the prognostic models constructed based on TCGA.

**Results:**

We identified that the prognostic 14-gene signature was significantly associated with the overall survival (OS) in the TCGA. In model A, patients in high- and low-risk groups showed the significantly different OS (P = 7.47 × 10^−9^, area under curve (AUC) 0.995) and the prognostic ability were also confirmed in testing sets (P=0.0098 and 0.037). The model B in training set was significant but failed in testing sets.

**Conclusion:**

The prognostic model which was constructed based on the prognostic 14-gene signature presented a high predictive ability for GBM. The 14-gene signature may have clinical implications in the subclassification of GBM.

## 1. Introduction

Glioblastoma (GBM) is the most aggressive diffuse and lethal malignancy in malignant gliomas [1]. To date, surgical resection followed by radiation therapy and chemotherapy is the frequently therapeutic intervention for GBM [2]. However, the therapy and prognosis of GBM remain dismal due to its invasive and aggressive behavior [3, 4]. GBM has a poor prognosis with relatively low survival and the five-year survival ratio is lower than 5% [5]. Therefore, it is important to further reveal novel therapeutic methods and underlying risk factors to improve the treatment and prognosis of GBM.

Poor prognosis with low relative survival rate is a major challenge for the treatment of GBM, and many risk factors have been identified to be associated with this outcome, such as age, gender, gene mutation, usage of drugs, and environment exposure [6]. Plenty of evidence indicates that many molecular biomarkers are significantly associated with the overall survival (OS) of GBMs and molecular features have been taken into account for the classification of GBM [7]. A centered classification of GBM based on the epidermal growth factor receptor (EGFR-) and platelet-derived growth factor receptor *α* has been built [8]. Besides, methylation status of the gene promoter for O6-methylguanine-DNA methyltransferase (MGMT), isocitrate dehydrogenase enzyme 1/2 mutation, was the prognostic molecules that have been fully confirmed [9, 10]. However, the prognostic model of GBM is still rarely reported.

In the current study, GBM prognostic genes were screened to construct a GBM prognostic model using the bioinformatics methods. Meanwhile, two datasets were utilized for validation. According to this, we aimed to explore a useful prognostic model for GBM and provide some useful insights in improving the prognosis of GBM patients.

## 2. Materials and Methods

### 2.1. Workflow of Construction of Prognostic Models

**Figure 1:**
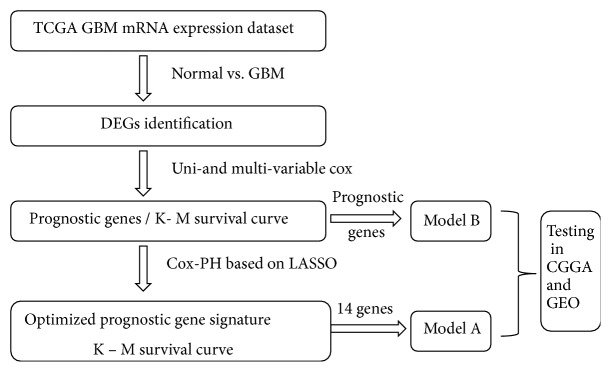
Workflow of construction of prognostic models for GBM.

A workflow of this study is shown in Figure 1. The gene expression profiles of GBM in the Cancer Genome Atlas (TCGA) were obtained and defined as the training set. Differently expressed genes (DEGs) between GBM and control groups were firstly identified. Then, the prognostic genes (Set X) are selected out by univariate and multivariate Cox regression analysis. After that, least absolute shrinkage and selection operator (LASSO) penalized Cox-Proportional Hazards (Cox-PH) model was used to further optimize the prognostic genes (Set Y). Next, two prognostic models were constructed based on the training set. Model A was based on the optimized prognostic genes (Set Y) which were identified by the Cox-PH regression analysis. We assumed that the number of genes used in model A was* Q*; Model B was based on the part of Set X. While, the number of genes applied in this model was* Q *rather than all the prognostic genes. GBM samples from the Chinese Glioma Genome Atlas database (CGGA, http://cgga.org.cn/) and GBM dataset GSE13041 were used to verify the model A and model B along with the Kaplan-Meier (K-M) survival analysis. Receiver operating characteristic (ROC) curve analysis was used to assess the prognostic gene signature.

### 2.2. Data Extraction and Grouping

Expression profiles of GBM were downloaded from the Cancer Genome Atlas database (TCGA, https://www.cancer.gov/about-nci/organization/ccg/research/structural-genomics/tcga), including 154 GBM tumor samples and 5 normal controls. All the samples were sequenced on the platform of Illumina HiSeq 2000 RNA Sequencing and utilized as the training set. Meanwhile, Chinese GBM expression profiles which are named as Part A were downloaded from the CGGA. A total of 128 GBM tissue samples involved in Part A were utilized as the testing set 1. Moreover, another GBM dataset GSE13041 downloaded from the Gene Expression Omnibus database (GEO, http://www.ncbi.nlm.nih.gov/geo/) was used as testing set 2. GSE13041 containing 191 GBM tumor tissue samples and was sequenced on the platform of Affymetrix Human Genome U133 Array.

### 2.3. Data Preprocessing and Differently Expressed Genes (DEGs) Screening

Based on the expression information provided by TCGA, edgeR (version 1.0.8, https://bioconductor.org/packages/release/bioc/html/edgeR.html) [11] in R 3.4.1 was utilized to screen DEGs between GBM and normal control samples with the thresholds of false discovery rate (FDR) <0.05 and |log fold change (FC)| >0.585. Meanwhile, for the GSE13041, raw data contained in CEL files were preprocessed by oligo (version 1.40.2, http://www.bioconductor.org/packages/release/bioc/html/oligo.html) [12] in R 3.4.1, including background correction and normalization. Then, according to the annotation information in platform, probes were annotated to gene symbols. In addition, for expression profiles in CGGA database presented in TXT format, genes were annotated using platform annotation profile. The gene expressions were transformed into logarithm of log2 by limma package (version 3.32.5, https://bioconductor.org/packages/release/bioc/html/limma.html) [13] in R 3.4.1 and were normalized using the median method.

### 2.4. Identification of Prognostic Genes

Basedon DEGs, survival package (version 2.41.3. http://bioconductor.org/packages/survival/) [14] in R 3.4.1 was applied to identify the association between the genes and patient's overall survival time (OS) by the univariate and multivariate Cox regression analysis. Genes were considered statistically significant when the P logrank values <0.05 were named as Set X. Then, the expression levels of prognostic genes were extracted from TCGA database. Bidirectional hierarchical clustering of these genes were conducted using the centered Pearson correlation method [15] provided by the heatmap package (version 1.0.8, https://bioconductor.org/packages/release/bioc/html/pheatmap.html) [16] in R 3.4.1. In addition, survival differences between the several clusters (based on the bidirectional hierarchical clustering analysis) were estimated by the K-M analysis with the log-rank test.

### 2.5. Further Analysis of the Prognostic Genes

Expression levels of the identified prognostic genes (Set X) were utilized as the input data to identify an optimal set of prognostic gene signature (named Set Y, the number of genes defined as* Q*) using the Cox-Proportional Hazards (Cox-PH) model [17], which was based on the LASSO-penalized regularization regression algorithm, provided by the penalized package (version 0.9.50, http://bioconductor.org/packages/penalized/) [18] in R 3.4.1. Lambda was used as the parameter in Cox-PH and obtained from 1000 times of cross-validation likelihood (CVL) cycle calculation. Besides, bidirectional hierarchical clustering was conducted by the centered Pearson correlation method, and survival difference between the several clusters was estimated by the K-M analysis with the log-rank test.

### 2.6. Construction and Verification of Prognostic Models

#### 2.6.1. Model A Based on the Optimized Prognostic Gene Signature (Set Y)

Using the optimized prognostic gene signature (Set Y) and coefficient of prognosis based on the Cox-PH method, model A was constructed and the prognosis index (PI) of each sample was computed. According to the median of PIs, the training dataset samples were divided into the high- and low-risk groups. Then, K-M survival curve analysis in R 3.4.1 survival package (version 2.41.3, http://bioconductor.org/packages/survival/) [19] was used to estimate the relations between model A and prognosis. Subsequently, model A was further verified in the two testing datasets and ROC curve analysis was used to assess the prognostic model.

#### 2.6.2. Model B Based on Part of the Prognostic Genes (Set X)

Based on logrank P value, the prognostic genes (Set X) were ranked in an ascending order, and the top* Q* genes of Set X were selected to calculate the coefficient of prognosis by multivariate Cox regression analysis. Based on the median of PIs, samples were divided into the high- and low-risk groups. Similar to the above, K-M survival analysis, ROC curve, and two testing datasets were also applied to assess and verify this model.

## 3. Results

### 3.1. DEGs Identified Based on the TCGA

**Figure 2:**
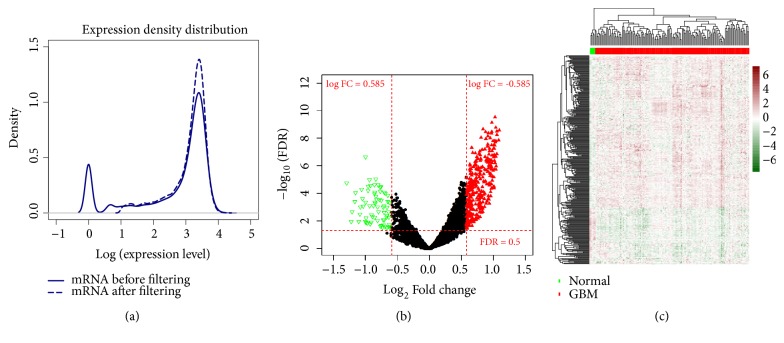
(a) The density distribution curve of gene expression values before and after filteration. (b) Volcano map. Red, green, and black dots indicate genes are upregulated, downregulated, and nonsignificant differentially expressed genes, respectively. (c) A bidirectional hierarchical clustering map based on 393 DEGs. Green and red sample bars represent normal control samples and tumor samples.

Gene expression levels of 159 GBM samples contained in TCGA were filtered and a total of 14626 genes were obtained with the median expression levels >1(Figure 2(a)). Based on the selective criteria, a total of 393 DEGs were identified between GBM and normal control groups, including 77 upregulated and 316 downregulated genes (Figure 2(b)). The detailed information (logFC, p value, and FDR) of 393 DEGs was listed in Supplementary Table 1. Then, bidirectional hierarchical clustering was conducted based on the 393 DEGs.  Figure 2(c) showed that the identified DEGs can significantly distinct tumor samples from the normal controls.

### 3.2. Identification of Prognostic Genes

**Figure 3:**
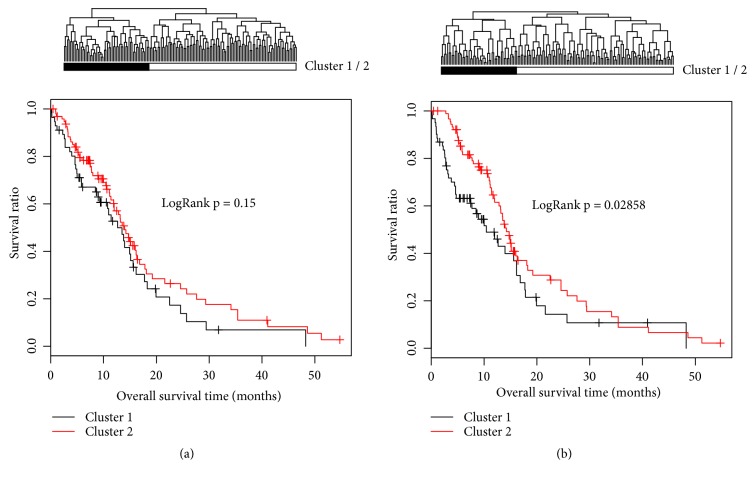
Clustering maps and Kaplan-Meier analysis based on the 43 GBM prognostic genes (a) or the optimized 14 prognostic gene signature (b). Cluster 1 is represented with black color, and cluster 2 is represented with white color. At the bottom, Kaplan-Meier curve analysis for cluster 1 and cluster 2.

Based on the 393 DEGs between GBM and normal controls, univariate and multivariate Cox regression analyses were performed, respectively. As a result, a total of 43 DEGs (named Set X) were significantly associated with the patient's OS. Then, samples were divided into cluster 1 (56 GBM samples,) and cluster 2 (96 GBM samples) according to the expression levels of 43 genes by bidirectional hierarchical clustering. In addition, the K-M curves assessed the OS of TCGA patients, and no significant difference was identified between cluster 1 and cluster 2 (Logrank P = 0.15) (Figure 3(a)).

### 3.3. Identification of the Prognostic 14-Gene Signature

**Figure 4:**
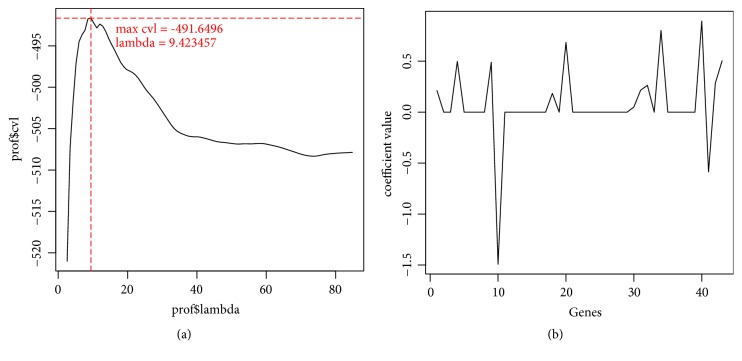
Screening the optimized prognostic genes by Cox-PH analysis. (a) *λ* value confirmation by CVL method. Cross of red pot line represented the selected *λ* = 9.423457. (b) Coefficient distribution of the optimized prognostic genes with *λ* = 9.423457.

With 1000 times of CVL calculation, CVL obtained the maximum value -491.6496 when *λ* = 9.42345 (Figure 4). The optimized prognostic gene signature contained 14 genes (Table 1) and the top 5 were* CPNE9*,* GUCA1A*,* INSL3*,* KHDRBS2*, and* KRT19*, respectively. The coefficient distribution is shown in Figure 3(b). The samples were divided into two significantly different clusters with the logrank P = 0.029.

### 3.4. Construction and Verification of Prognostic Models

#### 3.4.1. Model A Based on the Optimized Prognostic Genes (Set Y)

**Figure 5:**
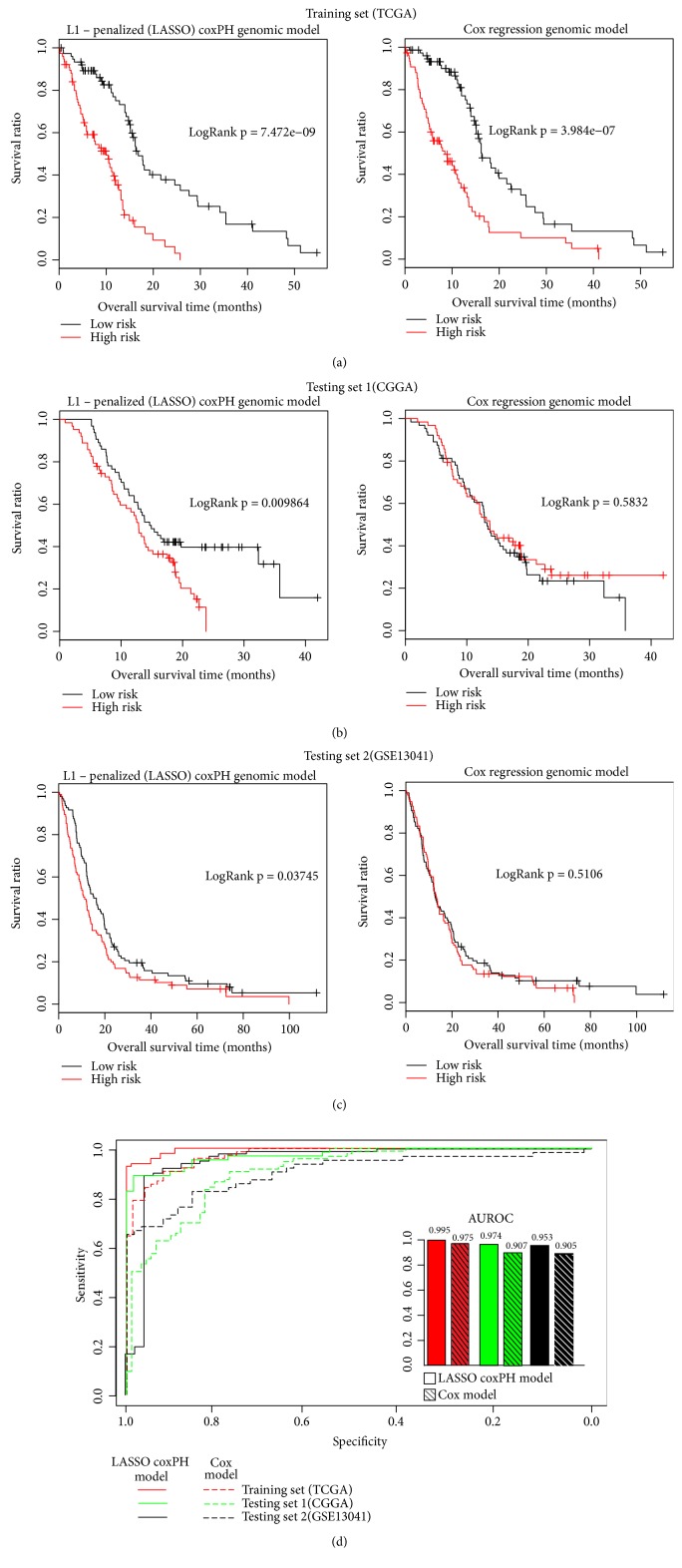
The K-M survival analysis and ROC curves of training and testing sets based on the prognostic model A and model B. (a) K-M curves in TCGA training set by model A (left) and model B (right). (b) K-M curves in CGGA testing set by model A (left) and model B (right). (c) K-M curves in GSE13041 testing set by model A (left) and model B (right). (d) ROC curves based on the PIs. In the figure, LASSO-Cox-PH genomic represents model A while Cox regression genomic model represents model B.

According to the Cox-PH coefficient, samples in the training set were divided into the high- (n = 76) and low-risk (n = 76) groups by the median of PIs = 10.96. The K-M survival analysis showed that the OS of patients in the low-risk group (15.94 ± 12.46 months) was significantly longer than that in the high-risk group (8.16 ± 5.89 months, P = 7.47 × 10^−9^, Figure 5(a) left). Moreover, this finding was also validated in the testing sets. For CGGA set, OS of patients with low PIs was significantly higher than that with high PIs (16.45 ± 8.93 versus 12.34 ± 6.52 months, P = 0.0098, Figure 5(b) left). Meanwhile, for GSE13041 set, the OS of patients in low- and high-risk groups were 21.99 ± 20.55 and 16.72 ± 17.91 months, respectively, with the P = 0.037 (Figure 5(c) left). Using PI as the forecast factor, ROC curve of PI was presented as Figure 5(d). The area under curve (AUC) was 0.995, 0.974, and 0.953, respectively, indicating that this model possessed a relative satisfied predicted ability.

#### 3.4.2. Model B Based on Prognostic Genes (the Top 14 Genes of Set X)

According to the logrank P value, we selected the top 14 genes of the 43 prognostic genes to construct model B. PI of each sample was calculated using the Cox multiple-factor regression analysis. Then, samples in the training set were divided into the high- and low-risk groups according to the median of PI = 5.87. K-M curves revealed that patients in the low-risk group presented longer OS time than that in the high-risk group (15.27 ± 11.22 versus. 8.86 ± 8.46, P = 3.98 × 10^−07^, Figure 5(a) right). In the CGGA and GSE13041 testing sets, patients were also divided into the high- and low-risk groups according to median PIs, but there was no significant difference in OS between the two groups (Figures 5(b) right and 5(c) right, P>0.05). Considering the validated results, model B was not suitable for the prognosis prediction of GBM despite the high AUC of ROC curves (Figure 5(d)).

## 4. Discussion

In this study, 43 GBM prognostic genes were identified based on the expression levels in TCGA by univariate and multivariate Cox regression analysis. Then, 14 out of them were further isolated as the optimized prognostic gene signature and to construct a prognostic model (model A), which presented a relative highly forecast ability for GBM. Model B was constructed based on the top 14 genes among of the 43 GBM prognostic genes. Compared with model B, model A showed a better predictive ability for GBM prognosis.

The technological advances in high-throughput sequencing and bioinformatics have enhanced the mining of the large volume of genetic data for disease. The gene expression profiles were widely used to predict the OS of the patients. Computationally, survival prediction is usually considered as a regression problem to model patients' survival time. The most common method is Cox regression models. Univariate and multivariate Cox regression were usually used to construct the prognostic models [20–22]. However, in this study, this method was not effective in CGGA and GSE13041. The Cox-PH model based on the LASSO is a semiparametric proportional hazards model where the covariates of the models explain the relative risks of the patients, termed hazard ratios [23]. Recently, increasing evidence has confirmed the availability of LASSO-Cox-PH model in survival analysis [24–26]. In this study, the prognostic model constructed by Cox-PH analysis showed a higher predictive ability both in training and testing sets.

The top five of the prognostic 14-gene signatures were* CPNE9*,* GUCA1A*,* INSL3*,* KHDRBS2*, and* KRT19*, respectively. Specifically,* KRT19* and* KHDRBS2* were two commonly reported genes in cancer.* KRT19* is the encoding gene of Keratin 19 and has been reported to play an important role in the development of cancer [27]. Saha et al. have identified that KRT19 interacts with *β*-catenin/RAC1 complex to regulate the properties of breast cancer cells, and knockdown of KRT19 promotes the proliferation, migration, invasion, and drug resistance [28]. Tang et al. have documented that KRT19 interacts with a novel biomarker linc00974 to promote the proliferation and metastasis of hepatocellular carcinoma [29]. Moreover, expression of KRT19 can be elevated by miR-200 to promote the metastasis of lung adenocarcinoma [30]. These findings indicated that KRT19 might play important roles in regulating the properties of cancer cells. However, studies of KRT19 are rarely reported in GBM. Considering this, it is important to reveal the mechanism of KRT19 in GBM.

*KHDRBS2*, the coding gene of KH RNA binding domain containing, signal transduction associated 2 (KHDRBS2), is reported to involve in several carcinogenesis. KHDRBS2 is muted in hepatitis B virus-induced hepatocellular carcinoma and closely related to the prognosis of HBV-induced hepatocellular carcinoma [31, 32]. Passon et al. have identified that KHDRBS2 is deleted in papillary thyroid carcinoma (PTC) and associated with the advanced PTC [33]. Moreover, in renal cell carcinoma,* KHDRBS2* is reported to form fusion part with* TFEB*, which is associated with the aggressive behavior of renal cell carcinoma [34]. These findings indicated that KHDRBS2 might play an important role in the development of carcinogenesis and might affect the prognosis of cancerous patients. In GBM, KHDRBS2 is significantly downregulated in two glioma cells lines LN229 and U373 cell lines and can be re-upregulated with different concentration of 5-aza-2-deoxycytidine, methylation inhibitor [35]. Moreover, a previous patent has demonstrated that the KHDRBS2 serves as a biomarker for the prognosis of GBM, and methylation status of* KHDRBS2 *correlates with the clinical survival outcome of GBM patients [36]. This might indicate that downregulation of KHDRBS2 may be involved in the development of GBM and could serve as a risk biomarker for GBM. In this study,* KHDRBS2 *was also identified to be associated with the prognosis of GBM. Combined with the other 13 genes, the predictive ability (AUC) could be achieved more than 0.95. These results indicated that this model might serve as a promising prognostic model for GBM.

The mutation of GUCA1A, guanylyl cyclase-activating protein 1, would lead to a severe dominantly inherited retinal degeneration (Cone dystrophy 3)[37]. INSL3 is an insulin-like hormone produced mainly in gonadal tissues and may be involved in the development of urogenital tract and female fertility. The mutation of INSL3 was related to the cryptorchidism [38, 39]. Besides, INSL3 increased the motility of thyroid carcinoma cells and high plasma INSL3 level was found in metastatic ovarian cancer, indicating that INSL3 was involved in the cancer development [40, 41]. The roles of CPNE9, GUCA1A, and INSL3 on GBM were rarely reported at present.

In conclusion, the prognostic model which was constructed by Cox-PH based on the prognostic 14-gene signature presented a relatively promising predictive ability for GBM. The 14 prognostic genes may have clinical implications in the subclassification of GBM.

## Figures and Tables

**Table 1:** The optimized prognostic 14-gene signature.

Features	Coef in coxPH	Hazard Ratio	p values
*Gene features*			
CCL7	0.4893	0.50749	0.011636
CPNE9	0.05016	3.33762	0.000737
GUCA1A	0.68468	2.74303	6.45E-07
HOXA11	-0.586	0.64545	0.030466
HOXC11	0.28422	2.47097	0.030154
HOXD11	0.50498	2.94223	0.042351
INSL3	0.21194	10.04671	7.48E-08
KHDRBS2	-1.4922	0.29589	0.000458
KRT19	0.4984	3.72613	7.20E-06
MEPE	0.26296	0.40042	0.001924
MLPH	0.21578	2.53208	0.00251
NELL1	0.8933	1.45172	0.025042
TBX5	0.18413	0.68823	0.022222
TMEM233	0.80243	2.97454	0.001871

## Data Availability

All data generated or analysed during this study are included in this published article.

## Supplementary Materials

Supplementary Materials Supplementary Table 1. The logFCp value and FDR of 393 DEGs between GBM and normal control samples.
